# Sharing Individual Participant Data from Clinical Trials: An Opinion Survey Regarding the Establishment of a Central Repository

**DOI:** 10.1371/journal.pone.0097886

**Published:** 2014-05-29

**Authors:** Catrin Tudur Smith, Kerry Dwan, Douglas G. Altman, Mike Clarke, Richard Riley, Paula R. Williamson

**Affiliations:** 1 North West Hub for Trials Methodology Research, Department of Biostatistics, University of Liverpool, Liverpool, England; 2 Centre for Statistics in Medicine, University of Oxford, Oxford, England; 3 All-Ireland Hub for Trials Methodology Research, Queen's University, Belfast, Ireland; 4 School of Health and Population Sciences, University of Birmingham, Birmingham, England; Cardiff University, United Kingdom

## Abstract

**Background:**

Calls have been made for increased access to individual participant data (IPD) from clinical trials, to ensure that complete evidence is available. However, despite the obvious benefits, progress towards this is frustratingly slow. In the meantime, many systematic reviews have already collected IPD from clinical trials. We propose that a central repository for these IPD should be established to ensure that these datasets are safeguarded and made available for use by others, building on the strengths and advantages of the collaborative groups that have been brought together in developing the datasets.

**Objective:**

Evaluate the level of support, and identify major issues, for establishing a central repository of IPD.

**Design:**

On-line survey with email reminders.

**Participants:**

71 reviewers affiliated with the Cochrane Collaboration's IPD Meta-analysis Methods Group were invited to participate.

**Results:**

30 (42%) invitees responded: 28 (93%) had been involved in an IPD review and 24 (80%) had been involved in a randomised trial. 25 (83%) agreed that a central repository was a good idea and 25 (83%) agreed that they would provide their IPD for central storage. Several benefits of a central repository were noted: safeguarding and standardisation of data, increased efficiency of IPD meta-analyses, knowledge advancement, and facilitating future clinical, and methodological research. The main concerns were gaining permission from trial data owners, uncertainty about the purpose of the repository, potential resource implications, and increased workload for IPD reviewers. Restricted access requiring approval, data security, anonymisation of data, and oversight committees were highlighted as issues under governance of the repository.

**Conclusion:**

There is support in this community of IPD reviewers, many of whom are also involved in clinical trials, for storing IPD in a central repository. Results from this survey are informing further work on developing a repository of IPD which is currently underway by our group.

## Introduction

Data that are collected on individuals who participate in a clinical trial are traditionally kept by the trial's research group or sponsor, with research utilising these data usually being undertaken by the same group. An increasing number of appeals have been made for these data to be made more widely available, accompanied by relevant documentation such as the protocol and any amendments (see references [1],[2],[3],[4] for example), to enable more complete and unbiased evidence syntheses, facilitate the identification of factors to personalise the delivery of medicine, and accelerate methodological research in clinical trials and evidence synthesis. Gøtzsche [2] describes a selection of examples, including the recent Tamiflu experience, that demonstrate the need for increased transparency and wider access to clinical trial data and documentation.

The BMJ recently announced a new policy on sharing clinical trial data which states that “from Jan 2013 trials of drugs and medical devices will be considered for publication only if the authors commit to making the relevant anonymised patient level data available on reasonable request” [5]. In 2011, The Cochrane Collaboration published a statement [6], which has recently been revised, supporting free access to all data from all clinical trials, and other journals have also issued data sharing policies [7],[8]. For example, PLoS journals [9] actively promote the linking of publications to ‘Dryad’ [10], an open access repository of data underlying peer-reviewed articles. The ‘All Trials’ initiative [11] is campaigning for the publication of all results from all clinical trials on all treatments, and their on-line petition had received more than 58,000 signatures by April 2014 from representatives of public funding bodies, journals, Royal Colleges, evidence based medicine organisations, charities, and pharmaceutical companies. The European Medicines Agency have issued their data sharing views [12] and are currently engaging in a consultation process, which includes pharmaceutical companies and academia, to develop an approach for sharing the individual participant data collected in trials used to support marketing approval applications. Further, public funding bodies such as the UK's Medical Research Council [13] and the National Institutes of Health in the USA [14] have specific data sharing policies that theoretically supports the open transfer of data collected on individual participants in the trials that they fund.

In anonline survey of 317 corresponding authors of clinical trials who had published their trials across six high impact journals in 2010 or 2011 [15], nearly three quarters of the respondents thought that sharing clinical trial data in repositories, or on specific request, should be a requirement. However, despite the wide support for sharing clinical trial data, there are significant challenges, and obtaining access to data is not as straightforward as it should be. For example, a survey by Savage [16] found that only 1 of 10 trials that had pledged an open data policy in their funding application actually provided the individual participant data when requested. Some of the challenges that have been identified previously include patient privacy issues, pharmaceutical companies' reluctance to share data with competitors, academic investigators protecting their publication potential, and a lack of resource to organise the data to be shared [17]. Despite these concerns, some trialists do make their data publicly available [18], and there are recent initiatives to develop repositories for trial data [10], [19] and standards for the public disclosure of data [20].

One advantage of central repositories of clinical trial data would be to accelerate the production of systematic reviews and meta-analysis of individual participant data (IPD), which are regarded as more reliable than other forms of systematic review [21]. The IPD approach to systematic reviews offers the opportunity to identify unpublished trials through collaboration with the original researchers, incorporate additional follow-up which can lead to improving the reliability of effect estimates, reduce bias by analysing on an intention to treat basis, minimise the possibility of within study selective reporting, standardise outcome definition across trials, and increase the potential to investigate subgroup effects. The main disadvantages of IPD reviews are that they require more resources, and rely on being able to gain access to participant level data from the included clinical trials to minimise the potential for data availability bias [22]. However, there are many examples in the literature of systematic reviews based on IPD and their number has increased from a few publications per year in the early 1990s to around 50 per year from 2005 [21]. IPD reviews often only address the main treatment efficacy question but many more questions could be explored using these datasets. For example, exploring multiple prognostic factors, assessing the effect of patient-level covariates (e.g. age, dose of drug, menopausal status) on treatment benefits and harms, and enhancing the ability to make reliable indirect comparisons and network analysis (see [23], [24], [25] for example) which should be considered as the bedrock for decisions when several treatments are available [26]. Since the IPD datasets have already taken a long time to prepare, with many issues identified and discussed with original researchers to reach the final ‘clean’ data used for re-analysis, there is a strong argument that these data should also be made available for research purposes, to maximise their value and research potential.

The Cochrane Collaboration IPD Meta-analysis Methods Group comprises people who are interested in the conduct of systematic reviews that include IPD, many of whom have participated in this type of research. We present results from a survey of all members of the Methods Group to explore their willingness to provide anonymised IPD (with the permission of the original researchers, as necessary) from previously conducted systematic reviews for central storage and management, to be made available for research projects, and to seek their opinions on the practicalities of doing so.

## Methods

A pilot questionnaire was developed by the research team and tested using a group of IPD reviewers attending a contributors' meeting of The Cochrane Collaboration. Feedback from this pilot phase was incorporated into an amended on-line questionnaire, which was developed using Surveygizmo [27]. A brief synopsis and hyperlink to the survey was emailed to all 71 members of the Cochrane IPD Meta-analysis Methods Group in March 2011, with three e-mail reminders sent to non-responders during April and May 2011. The questionnaire included 16 questions (Appendix S1) which would take an average of 10 minutes to complete. Ethical approval was not required from the University of Liverpool Research Ethics Committee as this project was considered a survey of current practice. Due to the online format of the survey, completion of the questionnaire was regarded as consent to participate and all data were anonymised. Free text responses were categorised by the lead author. All responses were summarised as percentages, with corresponding 95% confidence interval (Wilson score method).

## Results

**Figure 1:**
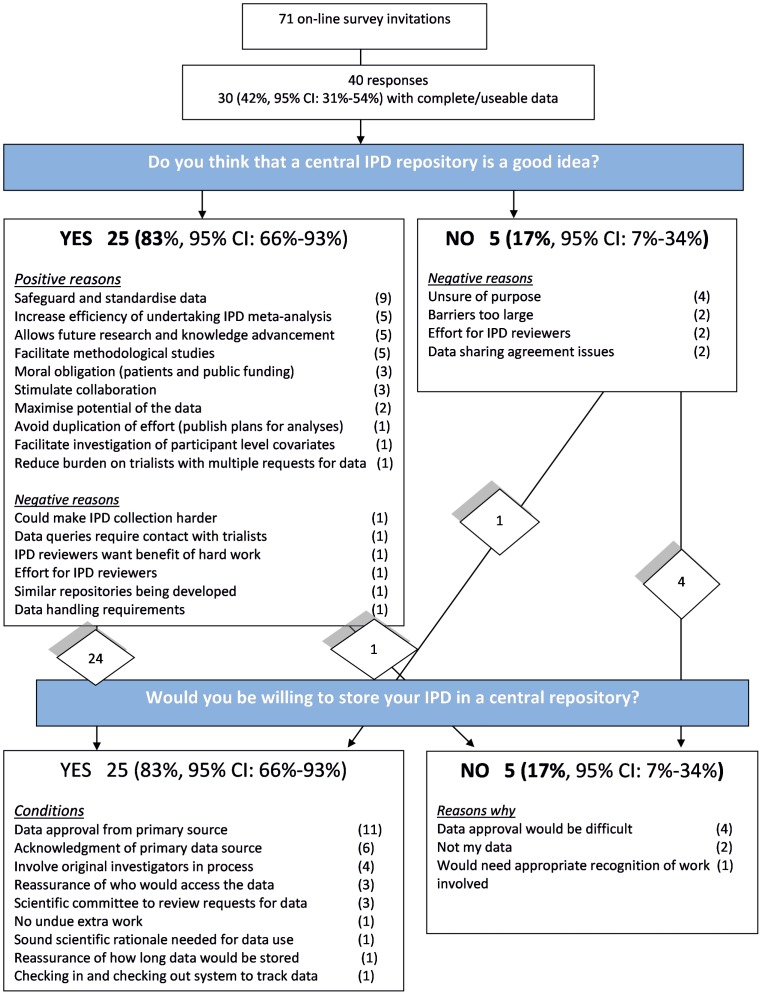
Flow diagram of survey responses.

Of the 71 people invited to participate, 40 responded (Figure 1). However, 10 of these responses were partial without any useable data and, therefore, the summary of results is restricted to the remaining 30 (response rate: 42%) complete responders.

Twenty two (73%) responders were from the United Kingdom, 6 (20%) from other European countries, 1 (3%) from Australia and 1 (3%) from Canada. The percentage of responders from each country is comparable to the percentage breakdown across the whole group of 71 contacts (60% UK; 28% other European countries; 2% Australia; 5% Canada; 5% other). Most of the responders had been involved in at least one IPD review (28 (93%)) and at least one randomised trial (24(80%)). Both IPD review and randomised trial involvement had been across a wide range of health areas including oncology, obstetrics and gynaecology, epilepsy, and surgery. Responders with randomised trial experience recorded a variety of roles within the trials, most as statisticians (9 (30%)), trial managers (6 (20%)) or investigators (4 (13%)). Responders' roles within systematic reviews also varied, with most stating that they had led, or contributed to the majority of the review.

Twenty five (83%, 95% CI: 66% to 93%) of the complete responders agreed that a central repository for IPD was a good idea (Figure 1). Among these 25 responders, the most commonly cited positive reasons (multiple reasons identified by some responders) were the need to safeguard and standardise data (9 (36%, 95%CI: 20% to 55%)), increase efficiency of undertaking IPD meta-analysis (5 (20%, 95% CI: 9% to 39%)), allow future research and knowledge advancement (5 (20%, 95% CI: 9% to 39%)), and facilitate methodological studies (5 (20%, 95% CI: 9% to 39%)). Some negative reasons were also highlighted by the 25 responders (Figure 1). Five of the 30 (17%, 95% CI: 7% to 34%) responders did not agree that a central repository for IPD was a good idea. For these 5, uncertainty of the purpose of such a repository (4 (80%, 95% CI: 38% to 96%)), barriers (2 (40%, 95% CI: 12 to 77%)), effort for the IPD reviewer (2 (40%, 95% CI: 12 to 77%)), and data release issues (2 (40%, 95% CI: 12 to 77%)) were the most common reasons for the negative response (Figure 1).

Unsurprisingly, most of the positive responders (24/25) would be willing to store their IPD in a central repository, whilst most of the negative responders (4/5) would not (Figure 1). Amongst the 25 willing responders, the most common requirements for depositing IPD in the repository were around gaining data approval (11 (44%, 95% CI: 27% to 63%)) and appropriate acknowledgement (6 (24%, 95% CI: 12% to 43%)) for the source of the IPD. The need to involve investigators in the process (4 (16%, 95% CI: 6% to 35%)), reassurances of who would access the data (3 (12%, 95% CI: 4% to 30%)), and the need for a scientific committee to review requests for data (3 (12%, 95% CI: 4% to 30%)) were also noted by more than one responder. The five responders who said that they would not store their data in a central repository noted that data approval would be difficult (3 (60%, 95% CI: 23% to 88%)) or that they could not store the data because it is not theirs (2 (40%, 95% CI: 12% to77%)).

Over a third of the 30 responders were uncertain about the data format and governance arrangements for a central repository (Table 1). The most common suggestion (9 (30%, 95%CI: 17% to 48%) responders) was for a simple data format to allow for different data types, e.g. comma separated variables (csv) or an Excel spreadsheet. Two (7%, 95% CI: 2 to 21%) responders noted the importance of password protection, 2 (7%, 95% CI: 2 to 21%) suggested that only a minimum dataset should be stored, and two (7%, 95% CI: 2 to 21%) suggested that a specific format for the data was not important if appropriate documentation accompanied the data (Table 1). When asked what governance arrangements would be expected, 10 (33%, 95%CI: 19% to 51%) responders stated that access to data should be restricted and require approval, 6 (20%, 95% CI: 10% to 37%) noted the importance of data security, 4 (13%, 95% CI: 5% to 30%) expected the use of an oversight committee, 4 (13%, 95% CI: 5% to 30%) would expect appropriate recognition for data owners, and 3 (10%, 95% CI: 3 to 26%) raised the issue of making sure that data were anonymised (Table 1).

**Table 1:** Views regarding the format of data and governance arrangements for a central repository of IPD.

	Number of responders (% and 95% CI)
**Q13. What format would you recommend for storing and accessing the IPD?** 1	
simple format to allow for different data types eg csv, excel	9 (30 (17 to 48))
password protected	2 (7 (2 to 21))
minimum dataset	2 (7 (2 to 21))
format not important	2 (7 (2 to 21)
SPSS	2 (7 (2 to 21))
Oracle, Stata, xml	1 (3 (0 to 17))
well documented data base including documentation for all errors and limitations	1 (3 (0 to 17))
not central storage but full description of data and where to access	1 (3 (0 to 17))
SAS	1 (3 (0 to 17))
relational database, in legacy format with metadata/documentation	1 (3 (0 to 17))
not sure/not answered	11 (37 (22 to 54))
**Q14. What governance arrangements would you expect?** 2	
restricted access requiring approval	10 (33 (19 to 51))
data security	6 (20 (10 to 37))
oversight committee	4 (13 (5 to 30))
recognition for data owners	4 (13 (5 to 30))
anonymised data	3 (10 (3 to 26))
clearly stated publication policy	2 (7 (2 to 21))
clear process required	2 (7 (2 to 21))
permission from data owners	2 (7 (2 to 21))
approval granted by data depositor	1 (3 (0 to 17))
as advised by QA	1 (3 (0 to 17))
all statutory requirements	1 (3 (0 to 17))
recognition for IPD principal investigator	1 (3 (0 to 17))
recognition for repository owners	1 (3 (0 to 17))
no governance required as data should be freely available	1 (3 (0 to 17))
not sure/not answered	11 (37 (22 to 54))

Responders could provide more than one reason so the numbers do not add to 30.

1 3 responders recorded two formats.

2 8 responders recorded two governance issues, 1 responder recorded three governance issues, 2 responders recorded four governance issues, 1 responder recorded five governance issues.

We also asked for specific advantages of a central repository, and obstacles (question 15 and 16). Some of these responses overlap with previous questions but we report them separately here for completeness (Table 2). The main reported advantages were to improve methodological research (7 (23%, 95% CI: 12% to 41%) responders), increase research using the data (5 (17%, 95% CI: 7% to 34%)), facilitate undertaking or updating IPD reviews (5 (17%, 95% CI: 7% to 34%)), and to safeguard the data (5 (17%, 95% CI: 7% to 34%)). The ability to undertake analyses across conditions or treatments, explore treatment effect modifiers, improve the quality of IPD reviews, increase the number of IPD reviews, increase collaboration, and transparency of research, were other noted advantages (Table 2). The main perceived obstacle was difficulty gaining permission from data owners, which was mentioned by 18 (60%, 95% CI: 42% to 75%) responders. Seven (23%, 95% CI: 12% to 41%) responders suggested that developing and maintaining the repository would be resource intensive and 3 (10%, 95% CI: 3 to 26%) felt that communicating the purpose of the repository could be an obstacle. Lack of acceptance from IPD reviewers if procedures for recognition were not in place, data coding issues, trust, difficulties reaching agreement on how data should be stored, and ethical issues, were other obstacles that were raised (Table 2).

**Table 2:** Views regarding the main advantages and main obstacles for a central repository of IPD.

	Number of responders (% and 95% CI)
**Q15. What do you expect to be the main advantages of creating a repository?** 1	
improving methodological research	7 (23 (12 to 41))
increase research using the data	5 (17 (7 to 34))
facilitate undertaking/updating of IPD reviews	5 (17 (7 to 34))
safeguard the data	5 (17 (7 to 34))
larger analyses across conditions or treatments	2 (7 (2 to 21))
Explore treatment effect modifiers	2 (7 (2 to 21))
sharing data	2 (7 (2 to 21))
improving quality of IPD reviews	2 (7 (2 to 21))
transparency of research	2 (7 (2 to 21))
increase collaboration between research groups	2 (7 (2 to 21))
increase number of IPD reviews	2 (7 (2 to 21))
extend repository to individual clinical trials	1 (3 (0 to 17))
sharing data	1 (3 (0 to 17))
not sure/not answered/‘see previous’	8 (27 (14 to 44))
**Q16. What do you expect to be the main obstacles to creating a repository?** 2	
difficulties gaining permission from data owners	18 (60 (42 to 75))
resource intensive to establish and maintain	7 (23 (12 to 41))
communicating the purpose of the repository	3 (10 (3 to 26))
lack of buy-in from IPD reviewers if procedures for recognition not in place	2 (7 (2 to 21))
data coding issues	2 (7 (2 to 21))
Trust	2 (7 (2 to 21))
difficulties getting agreement on how data should be stored	2 (7 (2 to 21))
ethical issues	2 (7 (2 to 21))
practical issues	1 (3 (0 to 17))
legal issues	1 (3 (0 to 17))
difficulties ensuring appropriate recognition to data owners	1 (3 (0 to 17))
not knowing the purpose	1 (3 (0 to 17))
ensuring data owners are involved with process	1 (3 (0 to 17))
governance issues	1 (3 (0 to 17))
not sure/not answered/‘see previous’	3 (10 (3 to 26))

Responders could provide more than one reason so the numbers do not add to 30.

1 8 responders recorded two advantages, 2 responders recorded three advantages, 1 responder recorded five advantages.

2 8 responders recorded two obstacles, 3 responders recorded three obstacles, 1 responder recorded four obstacles.

## Discussion

In this online survey of the members of the Cochrane Collaboration IPD Meta-analysis Methods Group, over 80% of responders agreed with the principle of creating a central repository for individual participant level data from randomised trials which had been collected for completed systematic reviews, with the same number of responders stating their willingness to share their IPD data sets. This survey complements the work by Rathi *et al* [15] which also showed strong support for data sharing: more than 70% of the corresponding authors of clinical trials published in a sample of high impact medical journals supported sharing de-identified data through data repositories or in response to individual requests. One of the strengths of our survey is that the majority of responders had been involved in both randomised trials and IPD reviews. They therefore represent the views of researchers who have been involved both in collecting and analysing data in individual studies (i.e. people who would deposit data from randomised trials) and in collecting and re-analysing data for evidence synthesis (i.e. end-users who would use the deposited data for new analyses). To our knowledge, this is the first survey to capture opinions about data sharing from this group of researchers who are able to provide their personal, all round view.

Some valid concerns were raised about the purpose, structure, governance arrangements and resource requirements for establishing and maintaining a central repository. The success of such an initiative would be fully dependent on gaining permission from the original clinical trialists who had provided their IPD for the systematic review. A minority of responders felt that this barrier, along with the increased effort and potential lack of appropriate recognition for the IPD reviewers, would preclude the success of such a facility. However, given that the clinical trialists have already agreed to share their data by contributing to the IPD review, they might be particularly amenable to the concept of a centralised repository. Therefore, as long as the appropriate governance and security measures are in place to protect and release the data to researchers, and as long as clinical trialists' concerns are addressed adequately (for example, through appropriate recognition to contributors), we do not believe that gaining permission from trialists will be a considerable barrier. Indeed, if it is not possible to gain permission from this group of trialists who are already engaged with data sharing for the purpose of IPD reviews, it is difficult to see how some of the data sharing visions, including those from an industry perspective that have been expressed in the literature recently(29), would ever be realised. Nevertheless, these potential barriers are worth exploring further. As an example of how better use could be made of IPD, authors of this paper have used the IPD from randomised trials of anti-epileptic drug trials in the late 1990s to (i) undertake a suite of head-to-head comparative meta-analyses [28]–[33], (ii) develop and apply methodology for a network analysis of IPD [23] which led to the estimation of comparative treatment effects that were not available from head-to-head randomised trials, (iii) inform the design of the largest ever trial in epilepsy [34], [35], (iv) contribute to the development of National Institute for Health and Clinical Excellence (NICE) guidance on epilepsy [36], (v) explore treatment effect modifiers [37] and (vi) as case studies to facilitate the development of methodological research in meta-analysis [24], [25], [38]–[42].

The issue of data sharing is topical at the moment, with several initiatives underway to make data from clinical trials more accessible. Of particular relevance is the European Medicines Agency's (EMA) draft policy on proactive access to clinical-trial data which was published in June 2013 for public consultation [43]. The policy, which has been welcomed by the research community, describes the EMA's plans to make future clinical trial data, including IPD, which is submitted to the EMA, available to external parties according to differing levels of control. There are some concerns and ethical implications that require further discussion in the clinical trials community and beyond but we hope that access to clinical trial data will become easier, and that the associated benefits will be realised. In the meantime, the appropriate storage and restricted release of IPD collected for systematic reviews is a logical and worthwhile venture and we are exploring issues of data release, participant confidentiality and governance of a central repository, which will then be developed and piloted. These plans will contribute to other ongoing work in the area of open access clinical trial data.

### Limitations of the study

This cross-sectional survey is limited to members of the Cochrane IPD Meta-analysis Methods Group and may not necessarily capture the opinion of IPD reviewers outside the Cochrane Collaboration. We do know that the majority of members are from non-commercial research institutes as it is these that are predominantly involved with IPD research. Therefore, it is possible that pharmaceutical industry representatives responsible for running clinical trials would have a different opinion to those expressed in this survey.

Although the survey was kept as simple as possible, required 10 minutes to complete, and was followed by three email reminders, only 42% of individuals we invited provided useable responses. Although disappointing, this is a typical response rate for online surveys [44] and is similar to the response rate of 46% achieved by the online survey of clinical trial data sharing by Rathi *et al* [15], which was despite their use of telephone reminders and the opportunity for responders to be entered into a prize draw to win one of five $100 gift certificates. Furthermore, when we compared the country of residence for responders against the country of residence for the full list of 71 members, this suggested that responders were a representative group. In addition, as the methods group is open for anyone to join it is entirely possible that some members are not actively involved with IPD research but may have at some time in the past expressed an interest in the topic. Members who have a strong opinion in favour or against the principle of data sharing are likely to have responded to ensure that their voice was heard. Therefore, we believe that non-responders were most likely to be those without a strong view on the subject, or those not actively involved with IPD meta-analysis research. Unfortunately, we have very little information regarding members' characteristics to verify this as these data are not routinely collected by the Cochrane methods group. We consider that the 30 responses are sufficient to provide initial discussion points on which to base our further work. It is also reassuring that our survey results are in keeping with a previous survey of clinical trialists [15].

The survey was originally launched in March 2011 and we recognise that responders' opinions may have changed. However, in response to a short internal consultation process to finalise the revised Cochrane Collaboration statement on access to trial data, a recent e-mail discussion within the Cochrane IPD Meta-analysis Methods Group identified very similar issues to those raised in our survey with the most contentious issue being whether data should be ‘completely open access’ or ‘restricted’ in some way. The topic of data sharing is incredibly fast moving with initiatives evolving very quickly. We believe that results from this survey, alongside other similar work that has been published can help to inform future research in this area. The questionnaire used open text fields, which were then categorised for presentation of results. The percentage of responders in each category should be interpreted cautiously. For example, only 3 (10%, 95% CI: 3 to 26%) responders specifically mentioned ‘anonymised data’ when asked about governance arrangements for the repository. However, it is highly likely that most responders would agree that anonymised data would be a critical element of this repository given that IPD meta-analyses are usually undertaken only with anonymised data. Therefore, the IPD reviewers may have assumed that this would also be the case within the repository, or it may be that they did not consider this issue to be relevant to the question about governance.

## Conclusions

Sharing clinical trial data is essential to accelerate research and increase transparency in trials, so that evidence-based decisions for patient care are informed by the highest quality and most complete data. Significant steps towards this ideal are already being made by funding bodies, regulatory authorities, pharmaceutical companies and medical journals which should strengthen future research. In the meantime, we need to consider how best to maximise the availability and impact of IPD that have already been collected for IPD systematic reviews and meta-analyses. If the emerging view is that providing IPD from individual trials should be an obligation, then it would follow that IPD from existing and future IPD meta-analyses should also be made available. A central repository for storing this IPD would be valuable and this survey has demonstrated support for this amongst IPD reviewers. Further research is now underway in regard to how this could be implemented and evaluated.

## Supporting Information

Appendix S1 (DOCX)
