# Supplementary material for: Sharing Individual Participant Data from Clinical Trials: An Opinion Survey Regarding the Establishment of a Central Repository
Source: PLoS One. 2014 May 29;9(5):e97886. doi: 10.1371/journal.pone.0097886 (PMC4038514; doi:10.1371/journal.pone.0097886)
Supplement: Appendix S1 — (DOCX) [file pone.0097886.s001.docx]

1. **Structure of the on-line survey**

**Welcome to the IPD repository questionnaire**

We (Doug Altman, Mike Clarke, Kerry Dwan, Richard Riley, Catrin Tudur Smith and Paula Williamson) are undertaking a survey to assess the feasibility of a central repository to store and manage access to individual participant data from systematic reviews of randomised controlled trials.

Through ensuring

 (i) adequate security measures, and

 (ii) involvement, with appropriate recognition of the data owners,

we believe that such a facility could have several significant benefits. We wish to obtain the opinions of as many people as possible and your help is very much appreciated. If you would like to complete this questionnaire and give us your comments, please click 'next'.

The survey will take around 10 minutes to complete and your progress can be saved and returned to at any point via 'save and continue' at the top of each page.

1. Name (optional):
2. Email address (optional):
3. Have you ever been involved with undertaking a randomised controlled trial before? Yes/No
4. Please describe the clinical area and your specific involvement:
5. Have you ever been involved with undertaking a systematic review before? Yes/No
6. Please describe the clinical area and your specific involvement:
7. Have you ever been involved with undertaking a systematic review using IPD before? Yes/No
8. Please describe the clinical area and your specific involvement:
9. Do you think a central repository for IPD is a good idea and why? Yes/No
10. Please say why:
11. Would you be willing to store your IPD in a central repository and would you have any conditions for doing so? No / Yes with conditions / Yes with no conditions
12. Please give your reasons / conditions:
13. What format would you recommend for storing and accessing the IPD?
14. What governance arrangements would you expect?
15. What do you expect to be the main advantages of creating a repository?
16. What do you expect to be the main obstacles to creating a repository?

**Thank you for taking our survey. Your response is very important to us.**
